# Molecular configuration-mediated thermo-responsiveness in oligo(ethylene glycol) derivatives attached on gold nanoparticles†

**DOI:** 10.1039/d1na00187f

**Published:** 2021-03-26

**Authors:** Kun Xiong, Hideyuki Mitomo, Xueming Su, Yier Shi, Yusuke Yonamine, Shin-ichiro Sato, Kuniharu Ijiro

**Affiliations:** Graduate School of Life Sciences, Hokkaido University Kita 10, Nishi 8, Kita-Ku Sapporo 060-0810 Japan; Research Institute for Electronic Science, Hokkaido University Kita 21, Nishi 10, Kita-Ku Sapporo 001-0021 Japan mitomo@es.hokudai.ac.jp; Global Institution for Collaborative Research and Education, Hokkaido University Kita 21, Nishi 11, Kita-Ku Sapporo 001-0021 Japan; Graduate School of Chemical Engineering and Sciences, Hokkaido University Kita 13, Nishi 8, Kita-ku Sapporo 060-8628 Japan; Faculty of Engineering, Hokkaido University Kita 13, Nishi 8, Kita-ku Sapporo 060-8628 Japan

## Abstract

Biomolecular systems actively control their local environment on a sub-nm scale *via* changes in molecular configuration from their flexible structures and derive emergent functions. Although this functional emergence based on local environmental control is attracting a great deal of attention in chemistry, it remains challenging to realize this artificially. Herein, we report the tuning of the thermo-responsive properties of oligo(ethylene glycol) (OEG) derivatives attached on gold nanoparticles *via* local environmental control not only by the hydrophobic moiety at their terminus but also by their molecular configuration. OEG-attached alkane thiol-modified AuNPs showed thermo-responsive assembly/disassembly in water through the hydration/dehydration of the OEG portions in a manner dependent both on the hydrophobicity at their terminus and the surface curvature of the core nanoparticles. Further, the assembly temperature (*T*_A_) was also tuned by ligand mixing with a non-thermo-responsive ligand with a shorter OEG length. Molecular dynamics simulations show that the distribution of the hydrophobic terminus in the normal direction along the gold surface varied in accordance with the surface curvature, indicating variations in molecular configuration. It is expected that a bent configuration could accelerate the thermo-responsiveness of OEG by allowing them greater accessibility to the hydrophobic terminus. Experimental and simulation results support the notion that local OEG density tuning by surface curvature or ligand mixing with a different OEG length leads to different degrees of accessibility to the hydrophobic terminus *via* changes in molecular configuration, promoting local environmental control-directed assembly temperature tuning.

## Abstract 2

Thermo-responsive properties of oligo(ethylene glycol) derivatives attached on gold nanoparticles were tuned *via* local environmental control not only by the hydrophobic moiety at their terminus but also by their molecular configuration.

## Graphical abstract

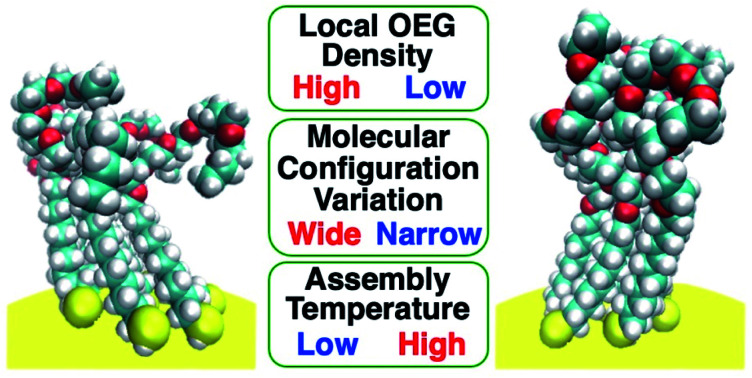

## Introduction

Molecules expand their functionality as polymers and/or supra-molecules through their connection, concatenation, or assembly. For example, in nature, peptides and proteins are produced by the connection of amino acids and show various properties which are not found in amino acids as monomers. Further, enzymes, which are proteins that take on sophisticated conformations *via* self-organization, exhibit high catalytic activities at an active site by controlling the local molecular states or atmosphere *via* molecular motion from their flexible structures.^1–3^ This functional emergence based on local environmental control on a sub-nm scale has been a focus of much attention in chemistry.^4–7^

Ethylene glycol-connected molecules, oligo and poly(ethylene glycol) (OEG and PEG), are particularly important synthetic molecules for medical and therapeutic applications due to their properties, such as neutral charge, water-solubility, and biocompatibility.^8–10^ PEG and its derivatives are attached to vesicles and nanoparticles to improve their dispersibility and retentivity in blood. Further, OEG-attached alkane thiols are used for the surface modification of biosensors to prevent undesired adsorption.^11,12^ PEG possesses not only bio-compatibility and bio-stealth properties but also thermo-responsiveness on hydration/dehydration states, like a poly(*N*-isopropyl acrylamide) (pNIPAM).^13,14^ Despite their common features, pNIPAM but not PEG is widely used as a thermo-responsive polymer.^15–17^ This is probably due to the difficulties associated with the tuning of thermo-responsiveness to preferable temperatures.^13,18^ On the other hand, OEG-derivative-grafted polymers^19,20^ and OEG-derivative-attached detergent assemblies (micelles)^21,22^ also show thermo-responsiveness as hydration/dehydration phenomena. The copolymerization of two OEG macromonomers of different chain-lengths with different degrees of hydrophilicity leads to the formation of thermosensitive copolymers with a tunable lower critical solution temperature (LCST).^23,24^ These kinds of thermo-responsive polymers with tunable LCST are now under the spotlight due to their potential therapeutic applications.^25^ Yet despite the enormous potential, there are no reports to date on their function evolution *via* control over their molecular configuration or the local state of the OEG portion.

Recently, gold nanoparticles (AuNPs) have attracted much attention in terms of potential therapeutic applications due to their plasmonic properties.^26–28^ However, when using AuNPs, surface modification remains an issue for the provision of stable dispersibility in solution. To this end, hydrophilic biocompatible polymers, such as PEG or OEG, are attached to their surfaces. Here, in terms of the surface modification of nanoparticles, there is one unmistakable fact: nanoparticle curvature controls the molecular conformation and chemical properties of the attached molecules by changing their local density or distances (in a range between 4–20 nm of AuNP diameter).^29–31^ Grzybowski *et al.* showed the unique possibilities for AuNP assembly regulated by electrostatics *via* location-specific charging on dumbbell-shaped nanoparticles as an effect of curvature.^31^ The shape of the core particle affords a way to control the molecular states within self-assembled monolayers and surface chemistries. This could be an advantage, not of polymers, but of small molecules or oligomers.^32^

**Scheme 1:**
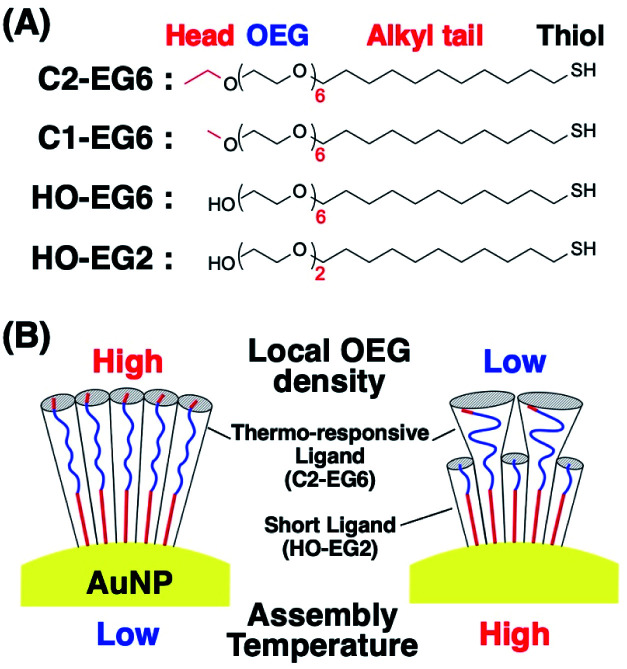
(A) Chemical structures of the surface ligands used in this study and (B) an illustration of the surface ligands attached on the AuNPs.

We have previously reported that gold nanoparticles (AuNPs) covered with OEG-attached alkane thiols show thermo-responsive assembly phenomena caused by hydrophobic interactions from the dehydration of the OEG portions.^33^ Importantly, their thermo-responsiveness shows a significant dependence on the core particle sizes. This size effect was confirmed as a surface curvature effect, not a volume effect, by using different sized rod-shaped gold nanoparticles.^34^ Further, we demonstrated the hierarchical self-assembly of rod-shaped gold nanoparticles based on curvature-dependent thermo-responsiveness.^34^ It seems that nanoparticle shape-based tuning of assembly temperatures (*T*_A_) is particularly useful in that it shows dramatic changes such as 67, 56, and 39 °C for gold nanoparticles of 3, 5, and 10 nm in diameter, respectively.^33^ However, as the exact control of NP size or curvature remains difficult, we could not precisely tune their *T*_A_, thus missing out on potential applications. In this study, we report a novel *T*_A_ tuning approach for AuNPs coated with OEG-derivatives by local molecular environmental control of thermo-responsive OEG-derivatives (C2-EG6) *via* mixing with short ligands (HO-EG2), which are not thermo-responsive under the applied experimental conditions, as molecular configuration-directed tuning (Scheme 1).

## Results and discussion

### Thermo-responsive properties of AuNPs coated with C1- or C2-EG6 ligands

First, we prepared thermo-responsive AuNPs by surface modification with a C1- or C2-EG6 ligand. The AuNP (diameter of 10, 15, and 20 nm) surfaces were modified with alkane thiol ligands *via* ligand exchange reaction in water according the method described in our previous paper.^33^ Citric acid and excess ligands were removed, and the solvent was replaced with 10 mM HEPES buffer (pH 8.0) *via* repetitive centrifugal purifications. Results for dynamic light scattering (DLS), zeta-potential, and extinction spectral analyses support the successful surface modification (Table S1 and Fig. S1†). Briefly, DLS measurements showed an increase of several nm in the hydrodynamic diameters of AuNPs by their surface modification with OEG-derivatives. The zeta-potential of the AuNPs changed from negative (*ca.* −30 mV in citric acid solution) for the citrate-protected AuNPs to weak negative (−8.7 ± 4.9 and −10.6 ± 3.1 mV in buffer) for C1- and C2-EG6-coated AuNPs (*d* = 15 nm), respectively. Extinction spectra showed a slight red-shift of the plasmonic peak from 517, 520, and 532 nm for citrate-coated to 522, 523, and 532 nm for C2-EG6-coated of AuNPs (10, 15, 20 nm), respectively, due to refractive index changes around the AuNPs.

**Fig. 1:**
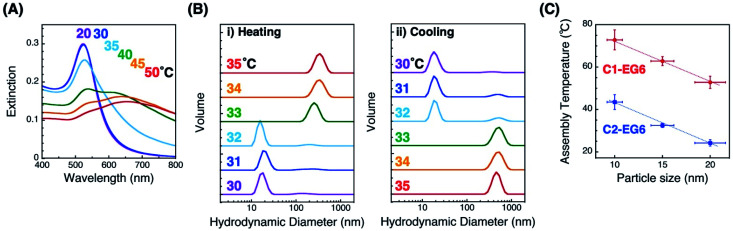
Thermo-responsive phenomena of AuNPs coated with C1- or C2-EG6 ligands. (A) Extinction spectra of C2-EG6-coated AuNPs (15 nm) during the heating process. (B) Size distribution of C2-EG6-coated AuNPs (15 nm) measured by DLS. Colored numbers in (A) and (B) represent solution temperature. (C) Assembly temperatures of 10, 15, and 20 nm AuNPs coated with C1-EG6 (red) and C2-EG6 ligands (blue). Error bars on the *y*-axis represent standard deviation determined from three independent experiments. Error bars on the *x*-axis represent the coefficient of variation shown on the data sheet from the company (BBI Solutions). Dotted lines are shown as a guide.

The thermo-responsive properties were then investigated by spectral and DLS analyses. Spectral changes indicated that C2-EG6-coated AuNPs (15 nm) showed thermo-responsive assembly between 30 and 40 °C, which is similar to our previous reports (Fig. 1A).^33^ Importantly, DLS measurements showed clear changes in size distribution responding to temperature changes in the heating and cooling processes (Fig. 1B). In the case of C2-EG6-coated AuNPs (15 nm), assembly/disassembly transition was observed between 32 and 33 °C. The assembly temperature for this experiment was, therefore, regarded as 32.5 °C. The temperature for their thermo-responsive assembly (*T*_A_) was next determined as 32.5 ± 1.0 °C from three independent DLS analyses on heating. In the same way, assembly temperatures (*T*_A_) of C2-EG6-coated AuNPs of different diameters (10 nm and 20 nm) were determined to be 43.5 ± 3.5 °C and 24.2 ± 1.5 °C, respectively (Fig. 1C blue and S2†). The *T*_A_ values of C1-EG6-coated AuNPs were also determined (Fig. 1C red and S3†). Fig. 1C clearly shows that the C2-EG6 ligand provided a lower *T*_A_ compared to the C1-EG6 ligand due to the hydrophobicity at its terminus, while the higher curvature from the smaller particle size provided a higher *T*_A_, findings which correspond to those reported in our previous paper.^33^

### Tuning of assembly temperature by hydrophobicity control *via* mixing of X-EG6 ligands with a different terminal group

**Fig. 2:**
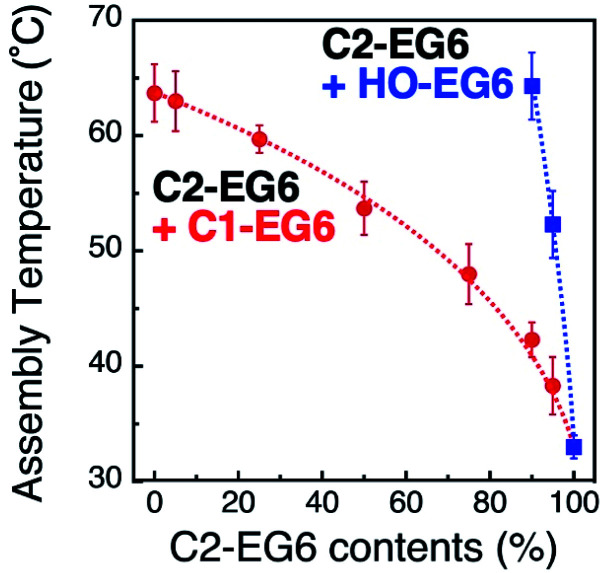
Assembly temperatures of 15 nm AuNPs coated with a mixture of C1- and C2-EG6 ligands (red) and C2- and HO-EG6 ligands (blue). Dotted lines are shown as a guide.

Precise tuning of the assembly temperature is an important issue for bio-related applications, particular tuning around 37 °C. Fig. 1C shows a 2 °C decrease in *T*_A_ with a 1 nm increase in diameter, suggesting that extreme control of AuNP size or shape can provide precise tunability. Although preparation techniques for nanoparticles have greatly advanced recently, size control on a sub-nm scale remains quite difficult.^35^ For example, the company (BBI Solutions) provides AuNPs of well-controlled size, which we used in this study, with a variation coefficient of within 8%. That is *ca.* 1 nm size distribution for 15 nm AuNPs, resulting in a 2 °C variation in *T*_A_. Thus, we performed assembly temperature tuning using ligand mixing as another approach using 15 nm AuNPs. First, we focused on the tuning of hydrophobicity by mixing surface ligands with different termini. For the surface modification of 15 nm AuNPs, we applied thermo-responsive ligands of C1-EG6 and C2-EG6 at various mixing ratios. These AuNPs showed obvious shifts in assembly temperature between 33 °C for 100% C2-EG6 and 63 °C for 100% C1-EG6 (Fig. 2 red and S4–S6†). As it is well-known that the LCST of thermo-responsive polymers, such as pNIPAm, is shifted to a lower temperature by the addition of hydrophobic moieties,^15^ this result is as expected. On the other hand, the great increase in assembly temperature observed on mixing with a HO-EG6 ligand, which has a neutral hydrophilic terminus, was impressive (Fig. 2 blue and S7†). Only 5% of HO-EG6 increased the *T*_A_ by 19 °C and 10% of HO-EG6 increased it by 32 °C from 33 °C (100% C2-EG6 ligands). In any case, it is possible to tune the assembly temperature by controlling the hydrophobicity at the terminus. The convex shape of these curves are similar to the clouding temperatures for binary mixture of various combinations of oligo(ethylene glycol)-based surfactants.^36^ In that report, the convex shapes were supported by Flory–Huggins theory extended to the binary surfactant system with an ideal mixing. Thus, these results are thought to be reasonable from the perspective of thermodynamics. Importantly, as the ligand mixing ratio is easily and precisely tunable, this is an effective approach.

### Tuning of assembly temperature by local OEG density control *via* mixing of OEG derivatives with a shorter OEG chain-length

Next, we focused on the effects of surface curvature (nanoparticle size) on the control of their thermo-responsive assembly. In our previous paper, we showed that the surface ligand density on AuNPs was determined to be *ca.* 5 molecules nm^−2^ by inductively coupled plasma atomic emission spectroscopy (ICP-AES) and was independent of their size and shapes.^33,34^ Surface curvature induces a different local density of the OEG portion at the outer surface, although their molecular density at the AuNP surface remains the same. Thus, we hypothesized their local density, which is related to the molecular free volume, is one of the key factors in tuning their assembly temperature. To tune their local density, here, we mixed the thermo-responsive ligand, C2-EG6, with a non-thermo-responsive ligand with a shorter OEG chain-length, HO-EG2 (Scheme 1).

**Fig. 3:**
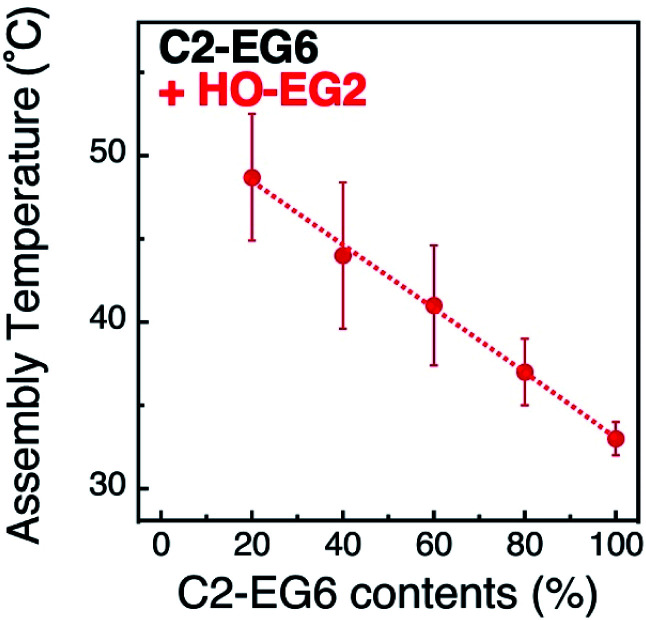
Assembly temperatures of 15 nm AuNPs coated with a mixture of C2-EG6 and HO-EG2 ligands. Dotted line is shown as a guide.

Assembly temperatures of 15 nm AuNPs coated with a mixture of C2-EG6 and HO-EG2 ligands was investigated in the same manner as described above (Table S4 and Fig. S8 and S9†). The plots of *T*_A_ by the ligand mixing ratios of C2-EG6 to HO-EG2 clearly indicate that the local molecular density tuned by mixing with a short-chain OEG ligand had a significant linear effect on *T*_A_ (Fig. 3). This increase in *T*_A_ by the replacement of C2-EG6 with HO-EG2 also includes changes in the hydrophobicity at the terminus. To discuss the effects of the tuning of local molecular density by mixing with a short-chain OEG ligand, comparisons of ligand replacement by C1-EG6, HO-EG6, and HO-EG2 are important. As the hydrophobic effect from the HO-EG2 portion is expected to be lower than that from the C1-EG6 portion and similar to that from the HO-EG6 portion, this moderate linear increase in *T*_A_ can be concluded to be mainly result from the tuning of local molecular density. Also, the difference in the correlation curves between the linear curve for C2-EG6/HO-EG2 (Fig. 3) and the convex curve for C2-EG6/C1-EG6 or HO-EG6 (Fig. 2) implies variations in their origin.

In these experimental results, the C2-EG6 content on the *x*-axis are shown as a ligand mixing ratio in the surface modification process as the real ligand content on the AuNP surfaces was unclear due to the experimental difficulties. It is reported that the difference in alkyl chain length, such as C12/C22 or C4/C18, induces domain structures in the self-assembled monolayer (SAM) formation of alkane thiols on flat surfaces.^37,38^ These results indicate that significant differences in the hydrophobic tails is a key factor in the phase segregation in SAMs. Further, there are several reports showing domain formations in the SAM on AuNP surfaces using small particles, indicating that a smaller size in diameter, particularly one less than 5 nm, also has an effect.^39,40^ Although we could not determined the real ligand contents on the surface, these reports suggest that there is no significant difference between the ligand mixing ratios in solution and ligand content on the AuNP surface.

### Molecular dynamics simulations

**Fig. 4:**
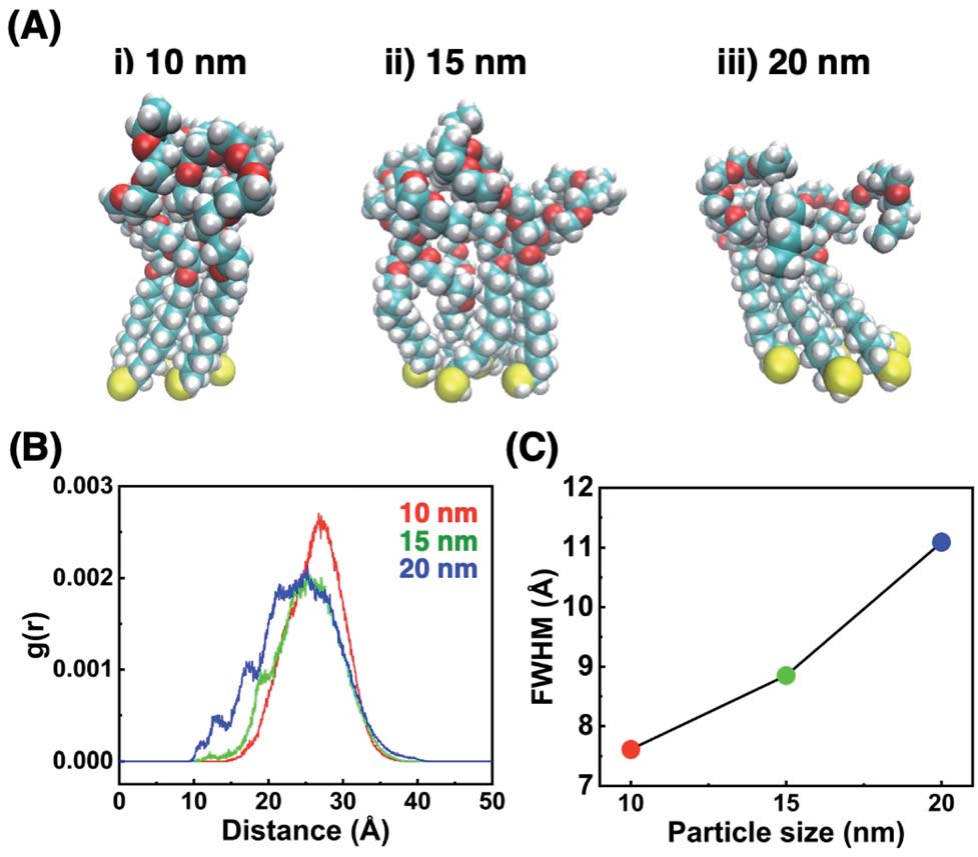
(A) Snapshots of seven C2-EG6 ligands in the vicinity of the surface center of 10- (i), 15- (ii), and 20 nm AuNPs (iii), (B) averaged RDF of the seven terminal carbon atoms against the corresponding sulfur atoms around the center of the gold surfaces, and (C) their FWHM on AuNPs of each diameter.

**Fig. 5:**
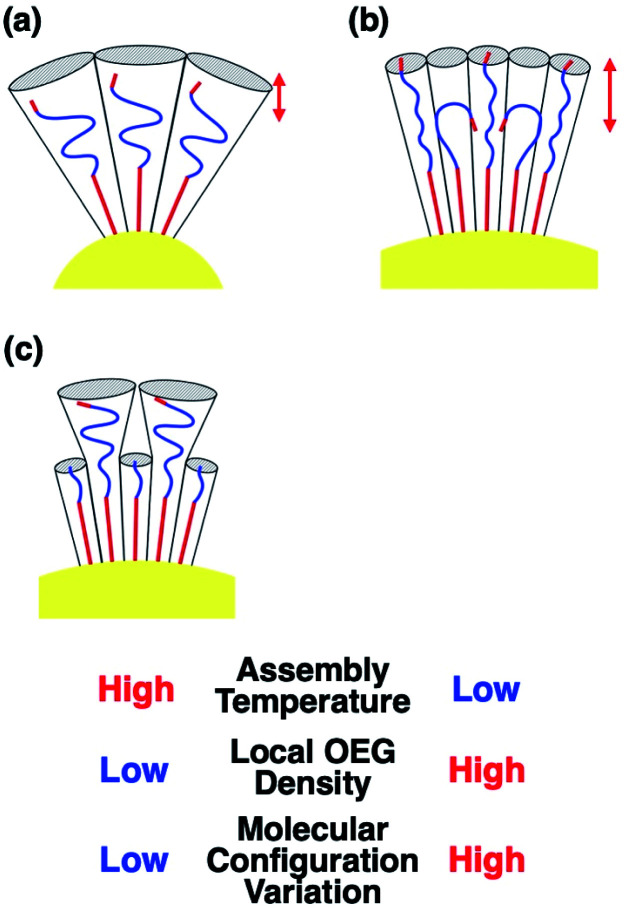
Schematic illustrations of the molecular configuration on AuNP surfaces. (a) C2-EG6 ligands on the surface with a high curvature. (b) C2-EG6 ligands on the surface with a low curvature. (c) C2-EG6 ligands mixed with HO-EG2 ligands on the surface with a low curvature. Red arrows represent the FWHM from the Fig. 4C, indicating a wide variations in their configuration.

**Fig. 6:**
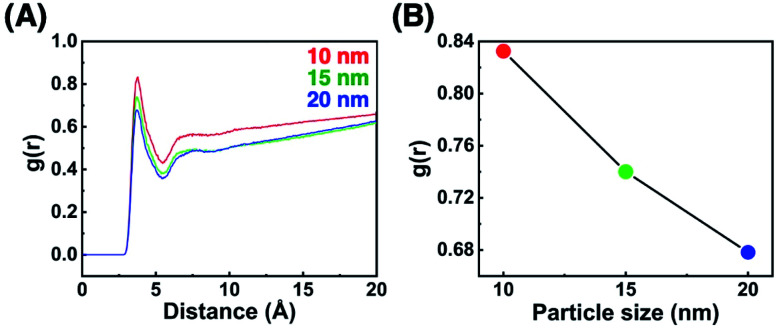
(A) The RDF of the terminal carbon atom against the oxygen atom of water molecules and (B) the intensities for the first peak in the RDF for each particle size.

To confirm that these *T*_A_ shifts result from changes in the local molecular state in the OEG portion of the surface ligands on curved surfaces, molecular dynamics (MD) simulations were performed. Briefly, 63–65 ligand molecules were attached on a curved surface in a limited space to tune the ligand density to *ca.* 5 molecules nm^−2^ (Fig. S10 and S11†).^33,34^ Then, calculations were performed using the AMBER14 program package with a GAFF force field.^41^ The molecular motion of the ligands around the central portion on the surface of 10-, 15-, and 20 nm AuNPs is shown in Fig. 4A and Movies S1–S3.† A flexible configuration of the OEG portion was observed compared to the alkyl chain tail portion. To clarify this molecular configuration, the radial distribution function (RDF) of the terminal carbon atom against the sulfur atom immobilized at the AuNP surface was analyzed for each molecules around the central portion (Fig. 4B for C2-EG6 and S12† for C1-EG6 ligands). The averaged full width at half maxima (FWHM) in each plot is shown in Fig. 4C. Interestingly, these data show that ligand molecules immobilized on larger AuNPs, which possess a lower curvature, possess a wider variations in the distribution of the hydrophobic terminus along the normal direction to the gold surface, indicating wider variations in molecular configuration, although it was expected that their distribution variations would be narrower due to lower molecular flexibility induced by structural restriction because of their smaller free volume (Fig. 5a and b). Our original idea was that the lower curvature provides a higher density of the OEG portion, leading to a relatively higher hydrophobic atmosphere around the OEG portion compared to that for a lower OEG density, and this difference in the surrounding conditions causes shifts in the *T*_A_. This still could be partially true. However, the MD simulation suggests that the hydrophobic (methyl or ethyl) group at the terminus is trapped into the OEG layer more often on surfaces with a lower curvature *via* a bent conformation than that on surfaces with a higher curvature. That is, a higher local OEG density promotes and stabilizes access of the hydrophobic terminus to the OEG portions due to the relatively higher hydrophobic atmosphere together with changes in the molecular configuration. Thus, the radial distribution function of the terminal carbon atom against water molecules was also analyzed for each molecules around the central portion (Fig. 6A). The shapes of their plots are similar to that of PEG calculated by another group.^42^ On the other hand, the intensity in the first solvation peak varied depending on particle size. The intensities for the first peak in the RDF for each particle size are plotted in Fig. 6B, which shows a clear relationship between *g*(*r*) values and particle sizes. Larger particles provided smaller solvation peak values, indicating that the carbon at the terminus is placed in a more hydrophobic environment. We also found that ligand molecules were sometimes trapped with an irregular configuration, in which the terminus was attached on the alkane thiols tails. These phenomena also support our notion described above. As this molecular bent configuration allows direct contact between hydrophobic terminus and the OEG portion, it can promotes their dehydration, causing a decrease in *T*_A_. That is, the molecular configuration derived from the local OEG density appears to be a critical factor. These MD simulation results suggested that different molecular configurations are provided by variations in the local OEG density due to different surface curvatures, thus inducing shifts in the thermo-responsivity of the OEG derivatives by differences in the degree of accessibility of the hydrophobic terminus.

Finally, we performed MD simulations for the mixtures of C2-EG6 and HO-EG2 ligands immobilized on a AuNP (15 nm) (Fig. S13†). Although the results include a large deviation due to the reduction in number of the thermo-responsive ligands (C2-EG6) due to the mixing with HO-EG2 ligands, a narrower FWHM for the distance distribution between the terminal carbon and sulfur atom was observed for the higher short-chain ligand (HO-EG2) content (Fig. S14A†). The intensities for the first peak in the *g*(*r*) of the terminal carbon atom against the oxygen atom of the water molecule increased with decreases in C2-EG6 content (increases in HO-EG2 content) (Fig. S14B†). These tendencies are the same as those observed for the curvature effect at a higher curvature. Further, both showed increase in assembly temperature (Fig. 1C and 3). These MD simulations support the notion that the assembly temperature of AuNPs modified with OEG-derivatives was tuned by the local OEG density and control of the ligand molecule configuration *via* mixing of short-chain ligands at various ratios instead of the controlling the AuNP size or curvature.

## Conclusions

In this study, the thermo-responsive properties of oligo(ethylene glycol) (OEG) derivatives attached on gold nanoparticles were tuned *via* the local environmental control not only by the hydrophobic moiety of their terminus but also by their molecular configuration. OEG-attached alkane thiol-modified AuNPs showed thermo-responsive assembly/disassembly through the hydration/dehydration of the OEG portions in a manner dependent on the hydrophobicity at the terminus. Further, the surface curvature of the core nanoparticles changes their responsive temperatures. Molecular dynamics simulations show that a hydrophobic terminus distribution along the normal direction to the gold surface varied in accordance with the surface curvature, indicating variations in the molecular configuration. A smaller curvature provides a higher local density, which promotes a bent configuration among the ligand molecules, probably due to the stabilization of the hydrophobic terminus in the relatively dense OEG moiety. This bent configuration could accelerate the thermo-responsiveness of the OEG portion *via* their greater degree of accessibility to the hydrophobic terminus. Importantly, the *T*_A_ was also tuned by ligand mixing with a non-thermo-responsive ligand with a shorter OEG chain-length. Although the MD simulations do not directly support this experimental result, experimental and simulation results on the curvature dependence of *T*_A_ support the notion that local OEG density tuning by surface curvature or ligand mixing with OEG of a shorter chain-length leads to differences in the degree of accessibility of hydrophobic terminus *via* changes in molecular configuration, thus promoting a local environment control-directed assembly temperature. As biomolecular systems actively control their local environment on a sub-nm scale *via* changes in their molecular configuration from their flexible structures and thereby derive emergent functions, our finding in this study provides a novel molecular design to the tuning of their functionality based on the molecular configuration control *via* local environmental tuning on a sub-nm scale as a biomimetic system, leading to opening a door for novel emergent functions.

## Experimental

### Materials

Citrate-protected AuNPs in aqueous solution (10, 15, and 20 nm in diameter) were purchased from BBI Solutions (UK). Surface ligands with a methyl, ethyl, and hydroxyl head, referred to as C1-EG6, C2-EG6, HO-EG6, and HO-EG2, respectively, were synthesized according to our previous paper (Scheme 1).^40,43^ All commercially available reagents were used without further purification. All solvents were purchased from Wako Pure Chemical Industries Ltd. (Japan).

### Surface modification of AuNPs

AuNPs coated with oligo(ethylene glycol) derivatives were prepared *via* a ligand exchange reaction. Briefly, 1 mL of citrate-coated AuNPs (10 nm, 15, and 20 nm) was concentrated by centrifugation (12 000*g* for 45 min) and the following removal of the supernatant (900 μL). The concentrated citrate-protected 10 nm AuNPs (94 μM, 100 μL), 15 nm AuNPs (23 μM, 100 μL), and 20 nm AuNPs (12 μM, 100 μL) were mixed with the thiolate ligand molecules including tris(2-carboxyethl)phosphine (TCEP) as a reductant. In this exchange reaction, the total ligand number was adjusted to 10 equivalent for the surface atoms on a AuNP.^44^ In the case of 10 nm AuNPs, the number of surface Au atoms was 4192/particle and the concentration of the ligand solution was 4.0 mM. In the case of 15 nm and 20 nm AuNPs, the number of surface Au atoms was 9585 and 17178/particle, respectively, and the ligand concentration for both was 2.0 mM. After the addition of ultrapure water up to 500 μL, the AuNPs were incubated for 48 h at 4 °C. Then, the ligand-exchanged AuNPs were washed 3 times by centrifugation (12 000*g* for 45 min), the removal of the supernatant (470 μL), and addition of 10 mM HEPES buffer (pH 8.0) up to 500 μL to remove the free thiol ligands.

### UV-vis-NIR spectroscopy

The UV-vis-NIR spectra of the AuNPs were measured using a V-770 UV-Visible/NIR Spectrophotometer with PAC-743R Automatic 6 position Peltier cell changer (JASCO Corp., Japan). The temperature-change measurements of the AuNP spectra were performed at each temperature with changes at a rate of 1 °C min^−1^ followed by a waiting time of 2 min.

### Dynamic light scattering (DLS) and zeta-potential measurement

The size and zeta-potential of AuNPs were measured using a Zetasizer Nano ZS (Malvern Panalytical Ltd, UK). The temperature-change measurements for the AuNPs sizes were performed at each temperature after a waiting time of 2 min. The assembly temperature was defined as the middle temperature between the temperature at which the size of the AuNPs showed a significant change and the highest temperature at which the AuNPs remaining dispersed.

### Simulation model

In the molecular dynamics (MD) simulations, partly due to the limitations in computing resources, we selected only a part of the particle surface for simulation in the absence of gold atoms. We employed this simplified model as the investigation of the local density dependence of OEGs on the curvature of the gold nanoparticle was our main computational purpose. For all the simulations, we used a cubic simulation box with a side length of 4 nm. The simulation boxes were set to intercept the top spherical cap of the nanosphere of gold nanoparticle with radius *R* (Fig. S10†).

### Numbers and initial configuration of the ligands on the surface of a sphere cap

According to the experimental condition, the ligand density on the particle surface was set at *ca.* 5 molecules nm^−2^. The spherical cap surface area formula was used to calculate the particle area in the box.*S* = 2π*RH*

In the above formula, *R* is the radius of the sphere, and *H* is the height of the spherical cap, which can be easily obtained by the Pythagorean theorem.

From the calculated surface area, the number of ligands was 65, 64, and 63 for 2*R* = 10 nm, 15 nm, and 20 nm, respectively.

To randomly plant the thiol end of the ligands on the surface of the sphere cap, a Packmol program^45^ was used in the modeling of the initial configuration of the ligand molecules. Furthermore, in order to make the oligomers evenly distributed on the surface of the spherical cap, the minimum distance between the molecules was set at 5 Å. An example of the configuration of C2-EG6 is shown in Fig. S11.†

The initial molecular structure and restrained electrostatic potential (RESP) charge of a single ligand were obtained by the AM1-bcc semi-empirical method using an antechamber program in Amber 14 (ref. 41) tools. The GAFF force field was used for the oligomer. The TIP3P model was used for the water molecule. About 130 000 water molecules were distributed in a cubic simulation box with a side length of 4 nm.

### Energy minimization, thermal equilibration, and MD calculation

Energy minimization at 0 K was carried out under constant-volume periodic boundary conditions by the steepest descent method. During minimization, all the sulfur atoms of each oligomer were fixed on the sphere cap with a binding force of kcal mol^−1^ Å^−2^. This binding force to the sulfur atoms was also used for the following calculations. The elevation of temperature from 0 to 300 K and thermal equilibration calculation was similar to those for energy minimization, using isometric periodic boundary conditions and constraining sulfur atoms. In the equilibrium and MD calculations, The SHAKE method was used to constrain all the chemical bonds containing hydrogen atoms and ignore the chemical bond forces. After the system reached a target equilibrium temperature at 300 K, an equilibration calculation was performed for 100 ps using a Langevin thermostat. After equilibration, the main MD simulations were carried out for 20 ns at 300 K. As for the above equilibrium calculation, the SHAKE method and the Langevin constant temperature controller were used. Throughout all the calculation, the interatomic cut-off radius of coulombic force was set at 9.0 Å. To confirm the reproducibility of the simulation, the same calculations were performed twice.

## Author contributions

H. Mitomo designed the research and experiments. K. Xiong and Y. Shi performed experiments. X. Su and S. Sato performed MD simulation. The manuscript was written with contributions from all authors. All authors have given approval to the final version of the manuscript.

## Conflicts of interest

There are no conflicts to declare.

## Supplementary Material

NA-003-D1NA00187F-s001

NA-003-D1NA00187F-s002

NA-003-D1NA00187F-s003

NA-003-D1NA00187F-s004
