# Supplementary material for: Molecular configuration-mediated thermo-responsiveness in oligo(ethylene glycol) derivatives attached on gold nanoparticles
Source: Nanoscale Adv. 2021 Mar 26;3(13):3762–9. doi: 10.1039/d1na00187f (PMC9418479; doi:10.1039/d1na00187f)
Supplement: NA-003-D1NA00187F-s001 [file NA-003-D1NA00187F-s001.pdf]

Electronic Supplementary Material (ESI) for Nanoscale Advances.
This journal is © The Royal Society of Chemistry 2021

Molecular Configuration-mediated Control of Thermo-responsiveness in Oligo(Ethylene Glycol) Derivatives Attached on Gold Nanoparticles

Xiong Kun,[†a] Hideyuki Mitomo,*[†bc] Xueming Su,[d] Yier Shi,[a] Yusuke Yonamine,[bc] Shin-ichiro Sato,*[de] and Kuniharu Ijiro*[bc]

[a] Graduate School of Life Sciences, Hokkaido University, Kita 10, Nishi 8, Kita-Ku, Sapporo 060-0810, Japan

[b] Research Institute for Electronic Science, Hokkaido University, Kita 21, Nishi 10, Kita-Ku, Sapporo 001-0021, Japan

[c] Global Institution for Collaborative Research and Education, Hokkaido University, Kita 21, Nishi 11, Kita-Ku, Sapporo 001-0021, Japan

[d] Graduate School of Chemical Engineering and Sciences, Hokkaido University, Kita 13, Nishi 8, Kita-ku, Sapporo 060-8628, Japan

[e] Faculty of Engineering, Hokkaido University, Kita 13, Nishi 8, Kita-ku, Sapporo 060-8628, Japan

[†] X.K. and H.M. contributed equally.

Experimental

Materials and Instruments.

All commercially available reagents were used without further purification. All solvents were purchased from Wako Pure Chemical Industries Ltd. (Japan). Thin-layer chromatography (TLC) was performed on glass-backed precoated silica gel plates (60F254, Merck & Co., Inc., USA). Cerium molybdate (10% Cerium (IV) Sulphate, 15%H_2SO_4 aqueous solution) was used as a coloring agent. Organic solvents were evaporated with an evaporator (TOKYO RIKAKIKAI Co., Ltd). Products were isolated by column chromatography on silica-gel (Kanto Chemical, neutral 60N, 40−50 μm). NMR spectra were recorded on a 400 MHz JEOL spectrometer. High-resolution ESI-MS spectra were measured with an Exactive LCMS Mass Spectrometer (Thermo Fisher Scientific Inc., Japan) by the Instrumental Analysis Division, Equipment Management Center Creative Research Institution, Hokkaido University.

AuNPs were analyzed by UV-Vis/NIR spectrophotometer (V-770, JASCO corp.) and dynamic light scatting (DLS) (Zetasizer Nano ZS, Malvern Panalytical).

Synthesis of HO-EG2

Scheme S1. Chemical synthesis of **HO-EG2** ligands

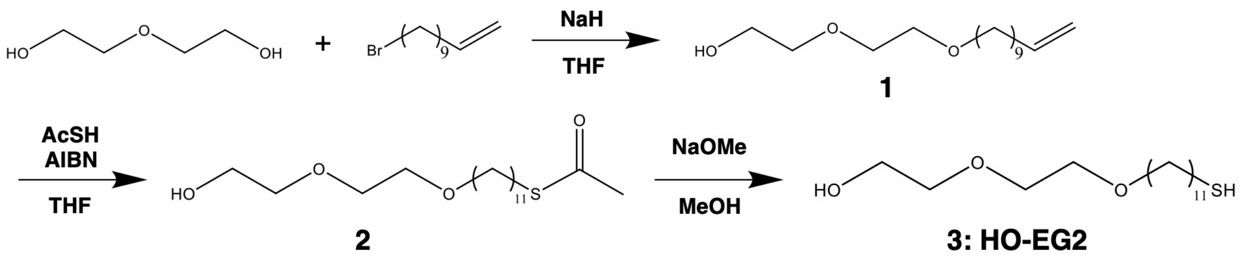

Synthesis of 3,6-Dioxa-16-heptadecen-1-ol; (1)

Diethylene glycol (14.9g, 140 mmol) was dissolved in THF (55 ml). The solution was cooled on ice and sodium hydride (60% in oil, 4.26 g, 106.4 mmol) and 11-bromo-1-undecene (6.53 ml, 28 mmol) added. The mixture was stirred at room temperature for 16 h, and then refluxed at 80°C for 16 h. After removal of the solvent, the residue was dissolved in solution (EtOAc/Hexane (1:1), 200 ml) and washed twice with a saturated NaCl solution. The organic layer was dried over anhydrous Na_2SO_4. The crude product was concentrated in vacuo and purified by flash chromatography on silica gel (Hexane/EtOAc (2:1)) to yield compound **1** (1.85g, 25%).

^{1}H NMR (400 MHz, $CDCl_3$): δ /ppm = 1.25-1.32 (m, 12H, alkyl chain), 1.56-1.60 (m, 2H, alkyl chain), 1.98-2.03 (q, 2H, -CH_2-*CH_2*-CH=), 2.48 (s, 1H, *HO*-), 3.42-3.72 (m, 10H, -*CH_2*-O-), 4.88-4.99 (m, 2H, -CH=*CH_2*), 5.74-5.84 (m, 1H, -*CH*=CH_2).

Synthesis of Ethanethioic acid, S-[11-[2-(2-hydroxyethoxy) ethoxy] undecyl] ester; (2)

Compound **1** (1.00 g, 3.87 mmol), thioacetic acid (1.52 g, 20.0 mmol), and 2,2'-azobisisobutyronitrile (0.636 g, 3.87 mmol) were dissolved in stabilizer-free dry THF (30 mL). The mixture was stirred for 3 h at 80 °C under a N_2 atmosphere. The mixture was evaporated under

a vacuum. The crude product was then concentrated in vacuo and purified by flash chromatography on silica gel (Hexane/EtOAc (1:1)) to yield compound **2** (0.65 g, 50 %) as a clear syrup.

^{1}H NMR (400 MHz, $CDCl_3$): δ /ppm = 1.26-1.32 (m, 14H, alkyl chain), 1.57-1.60 (m, 4H, alkyl chain), 2.30-2.32 (s, 3H, -S-CO-CH_3), 2.84-2.87 (t, 2H, -CH_2-CH_2-S-), 3.44-3.49 (t, 2H, HO-CH_2-), 3.58-3.73 (m, 8H, -CH_2-O-).

Synthesis of 2-[2-[(11-Mercaptoundecyl) oxy] ethoxy] ethanol; (3) HO-EG2

Compound **2** (0.65 g, 1.94 mmol) and sodium methoxide (powder, 0.52g, 9.7 mmol) were dissolved in 20 mL MeOH, and the mixture was stirred overnight at 30 °C. After adding HCl aq. until the solution pH<7, the solvent was removed under a vacuum. The residue was dissolved in EtOAc and washed twice with sat. NaCl solution. The organic layer was dried over anhydrous Na_2SO_4. The crude product was concentrated in vacuo and purified by flash chromatography on silica gel (Hexane/Acetone (2:3)) to yield compound **3** (0.07g 12.5%) as a clear syrup.

^{1}H NMR (400 MHz, $CDCl_3$): δ /ppm = 1.26-1.38 (m, 14H, alkyl chain), 1.55-1.62 (m, 4H, alkyl chain), 2.49-2.54 (q, 2H, -CH_2-SH), 3.44-3.48 (t, 2H, HO-CH_2-), 3.57-3.73 (m, 8H, -CH_2-O-).

HR-MS (ESI): calcd for $C_{15}H_{32}O_3SNa$ $[M+Na]^+$ 315.19644, found 315.19615.

Table S1. Hydrodynamic diameter and zeta-potential of AuNPs before and after surface modification with a **C1-** or **C2-EG6** ligand.

10 nm AuNP	Zeta-Potential (mV)	S.D.	Size*[1] (nm)	S.D.
Citric Acid	-26.9	2.5	10.4	0.2
C1 100%	-7.5	2.1	13.6	0.6
C2 100%	-8.0	0.5	13.7	0.6

15 nm AuNP	Zeta-Potential (mV)	S.D.	Size*[1] (nm)	S.D.
Citric Acid	-31.3	1.4	15.1	0.6
C1 100%	-8.7	4.9	22.6	6.3
C2 100%	-10.6	3.1	19.7	0.3

20 nm AuNP	Zeta-Potential (mV)	S.D.	Size*[1] (nm)	S.D.
Citric Acid	-37.7	2.5	19.2	0.2
C1 100%	-9.0	1.9	50.5	50.3
C2 100%	-14.3	0.6	26.7	4.0

*[1] Size was determined as the main peak size in the DLS size distribution by volume.

Large size numbers among the 20 nm-AuNPs could result from a partial aggregation due to their hydrophobicity.

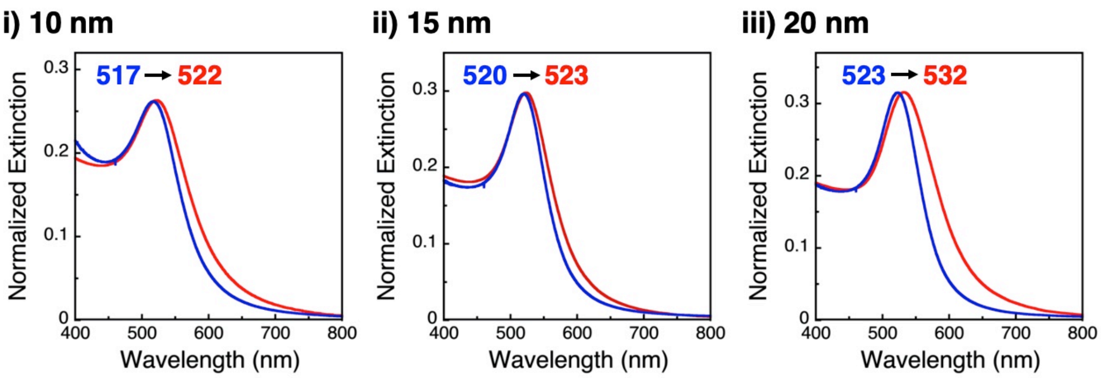

Figure S1. Extinction spectra of AuNPs of 10 nm (i), 15 nm (ii), and 20 nm in diameter (iii) before (blue) and after surface modification with **C2-EG6** ligands (red). The numbers indicate their peak wavelength.

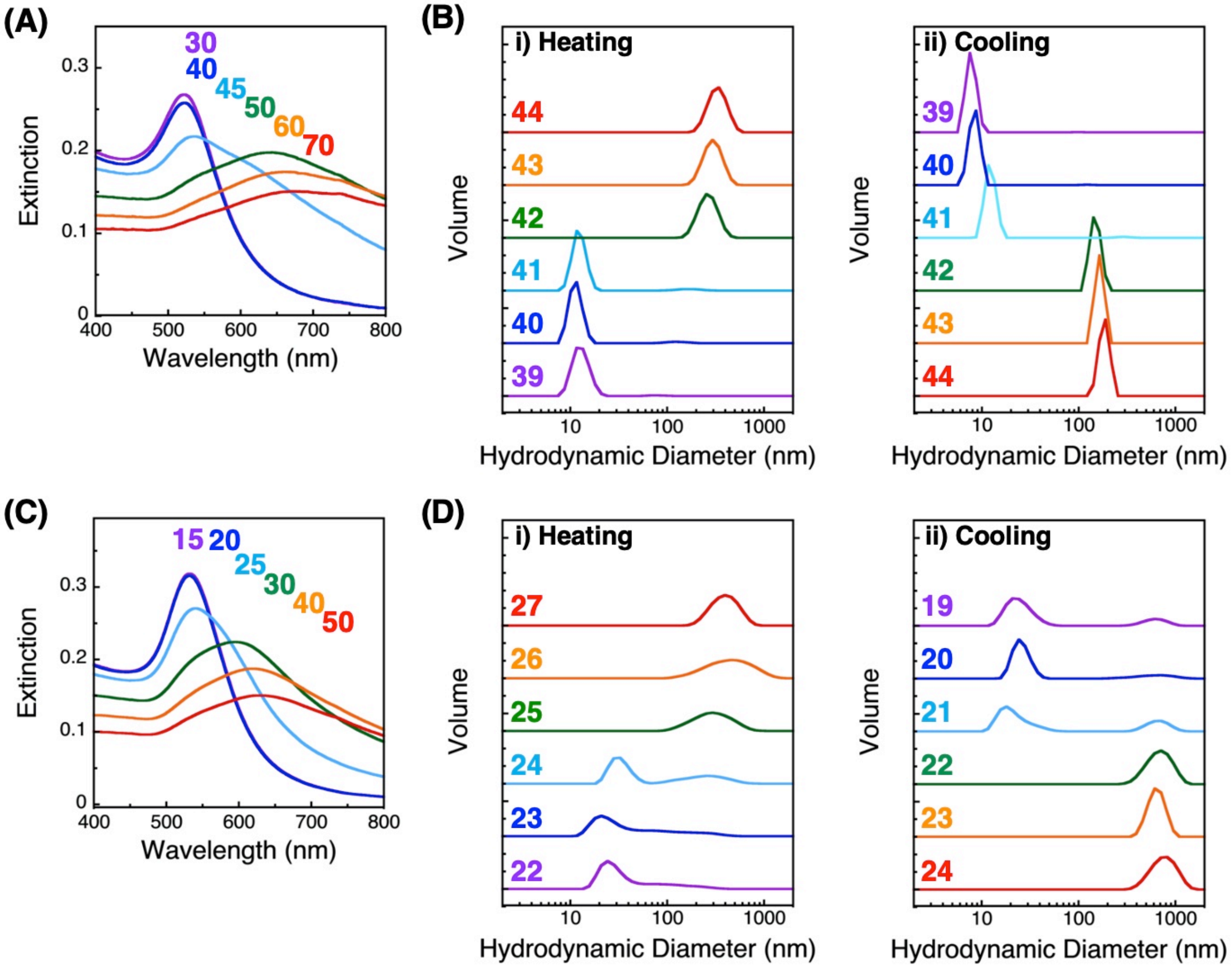

Figure S2. Extinction spectra (A)(C) and size distribution (B)(D) of **C2-EG6** ligand-modified AuNPs of 10 nm (A)(B) and 20 nm in diameter (C)(D).

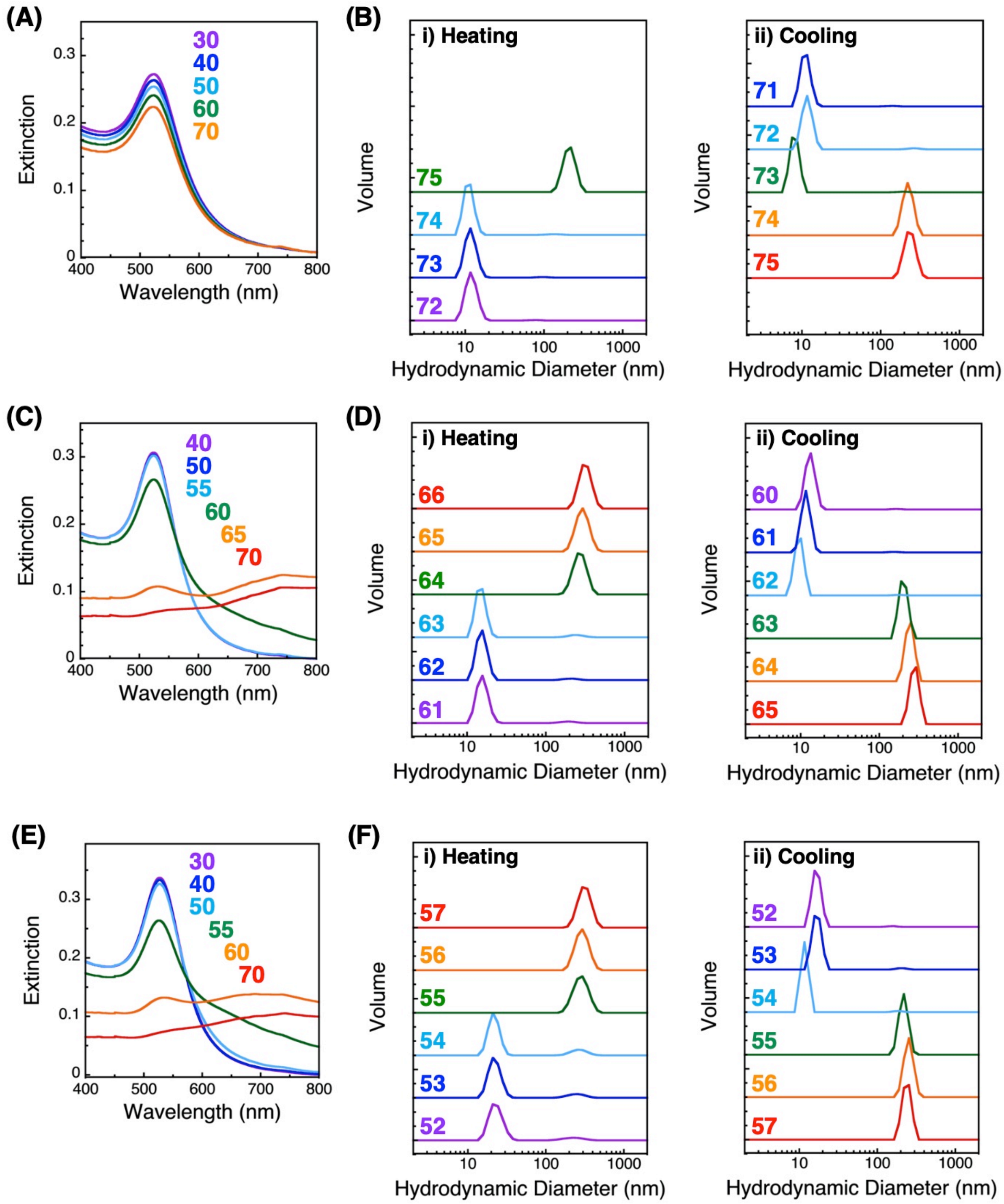

Figure S3. Extinction spectra (A)(C) and size distribution (B)(D) of **C2-EG6** ligand modified AuNPs of 10 nm (A)(B), 15 nm (C)(D), and 20 nm in diameter (E)(F).

Table S2. Hydrodynamic diameter and zeta-potential of AuNPs before and after surface modification with a mixture of **C2-EG6** and **C1-EG6** ligands.

**C2-EG6 : C1-EG6 Ligand Ratio**	Zeta-Potential (mV)	S.D.	Size*[1] (nm)	S.D.
95 : 5	-6.1	1.5	22.5	3.6
90 : 10	-7.0	2.1	20.5	1.5
75 : 25	-5.7	1.3	18.3	0.2
50 : 50	-6.7	0.4	23.1	4.6
25 : 75	-5.5	1.5	26.3	6.2
5 : 95	-6.2	1.0	22.4	6.2

*[1] Size was determined as the main peak size in the DLS size distribution by volume.

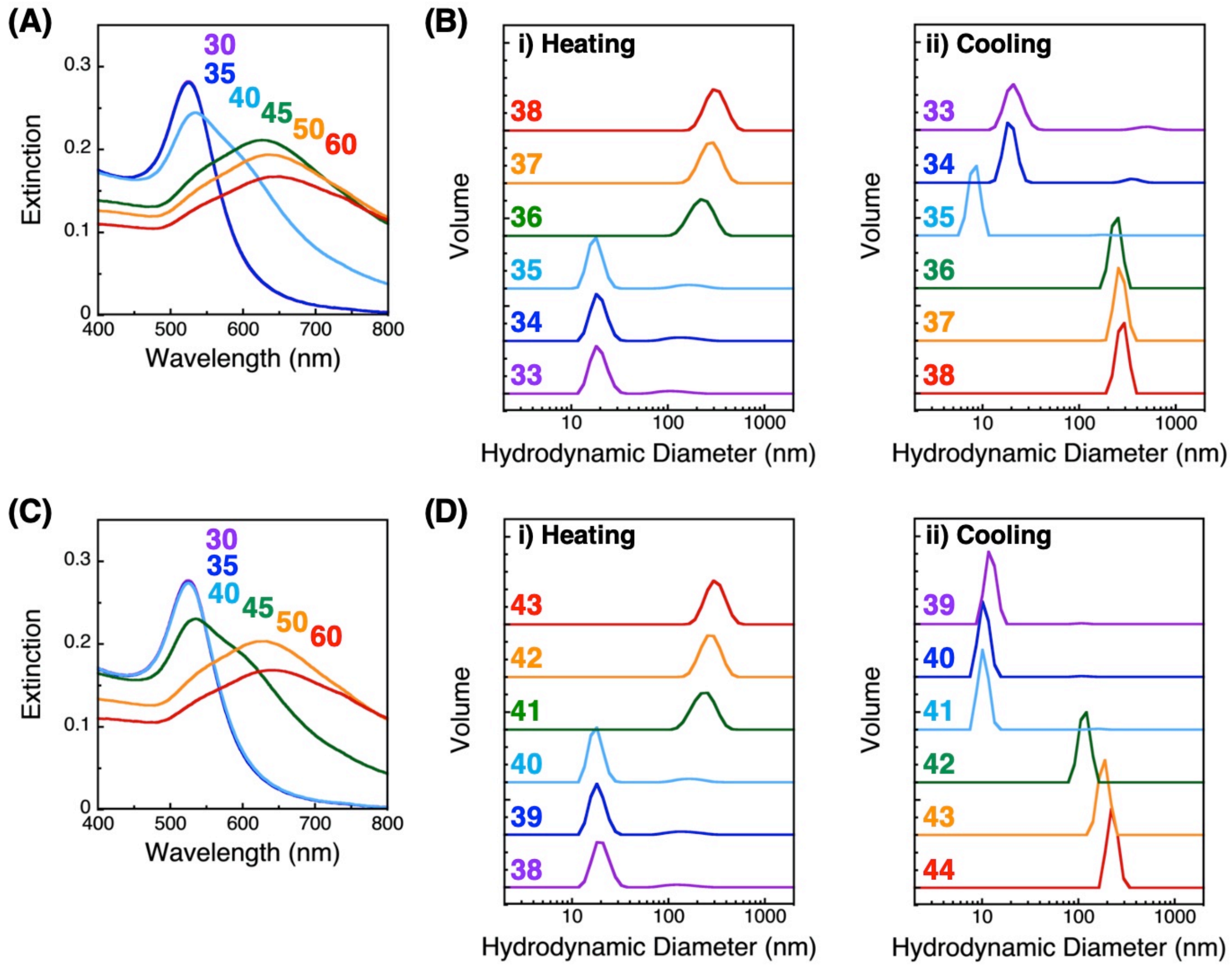

Figure S4. Extinction spectra (A)(C) and size distribution (B)(D) of 15-nm AuNPs coated with a mixture of **C1-EG6** and **C2-EG6** ligands. (A)(B) AuNPs modified with **C2-EG6** (95%) + **C1-EG6** (5%). (C)(D) AuNPs modified with **C2-EG6** (90%) + **C1-EG6** (10%).

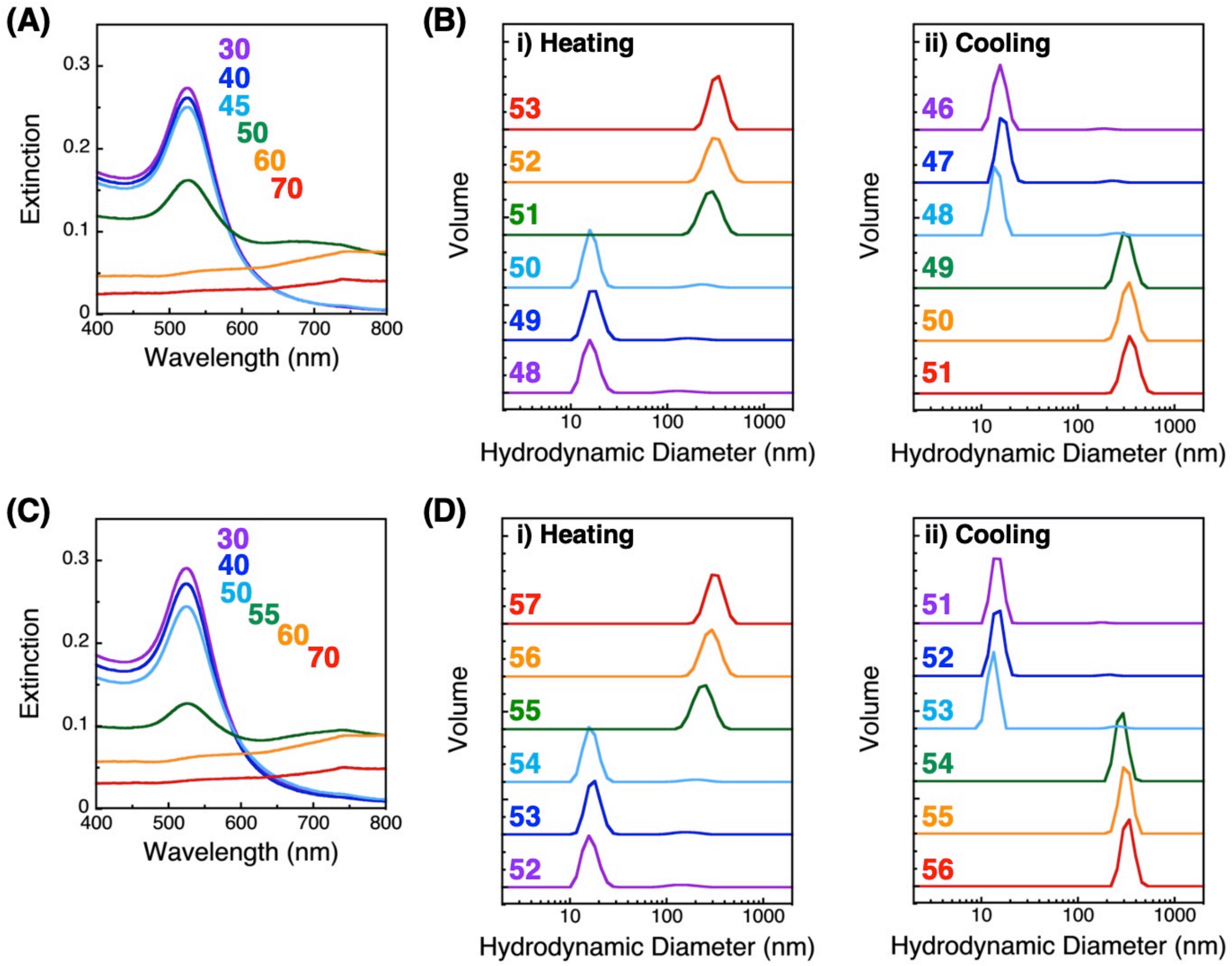

Figure S5. Extinction spectra (A)(C) and size distribution (B)(D) of 15-nm AuNPs coated with a mixture of **C1-EG6** and **C2-EG6** ligands. (A)(B) AuNPs modified with **C2-EG6** (75%) + **C1-EG6** (25%). (C)(D) AuNPs modified with **C2-EG6** (50%) + **C1-EG6** (50%).

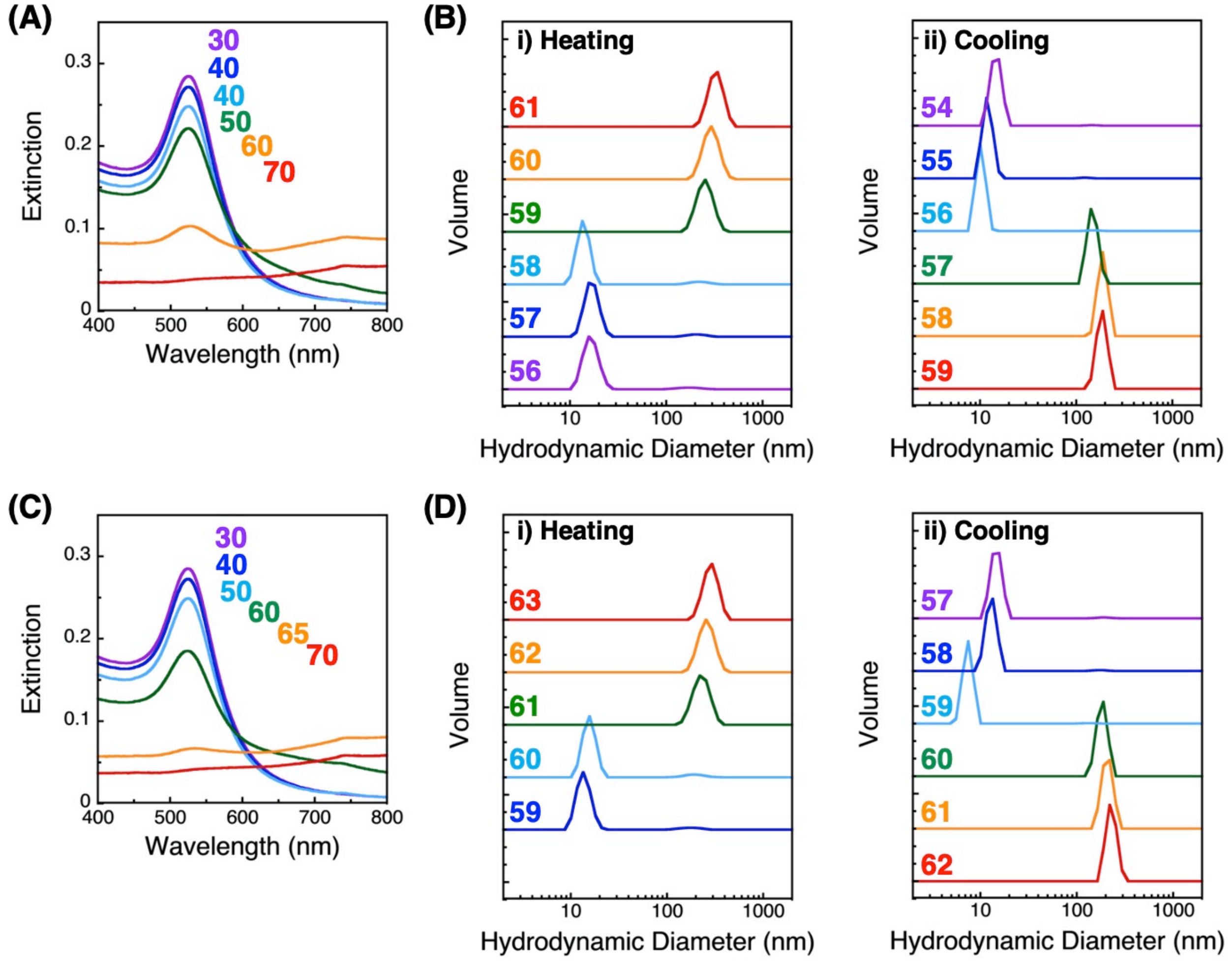

Figure S6. Extinction spectra (A)(C) and size distribution (B)(D) of 15-nm AuNPs coated with a mixture of **C1-EG6** and **C2-EG6** ligands. (A)(B) AuNPs modified with **C2-EG6** (25%) + **C1-EG6** (75%). (C)(D) AuNPs modified with **C2-EG6** (5%) + **C1-EG6** (95%).

Table S3. Hydrodynamic diameter and zeta-potential of AuNPs before and after surface modification with a mixture of **C2-EG6** and **HO-EG6** ligands.

**C2-EG6 : HO-EG6 Ligand Ratio**	Zeta-Potential (mV)	S.D.	Size*[1] (nm)	S.D.
95 : 5	-6.9	1.3	23.7	7.3
90 : 10	-6.4	0.5	22.3	4.6

*[1] Size was determined as the main peak size in the DLS size distribution by volume.

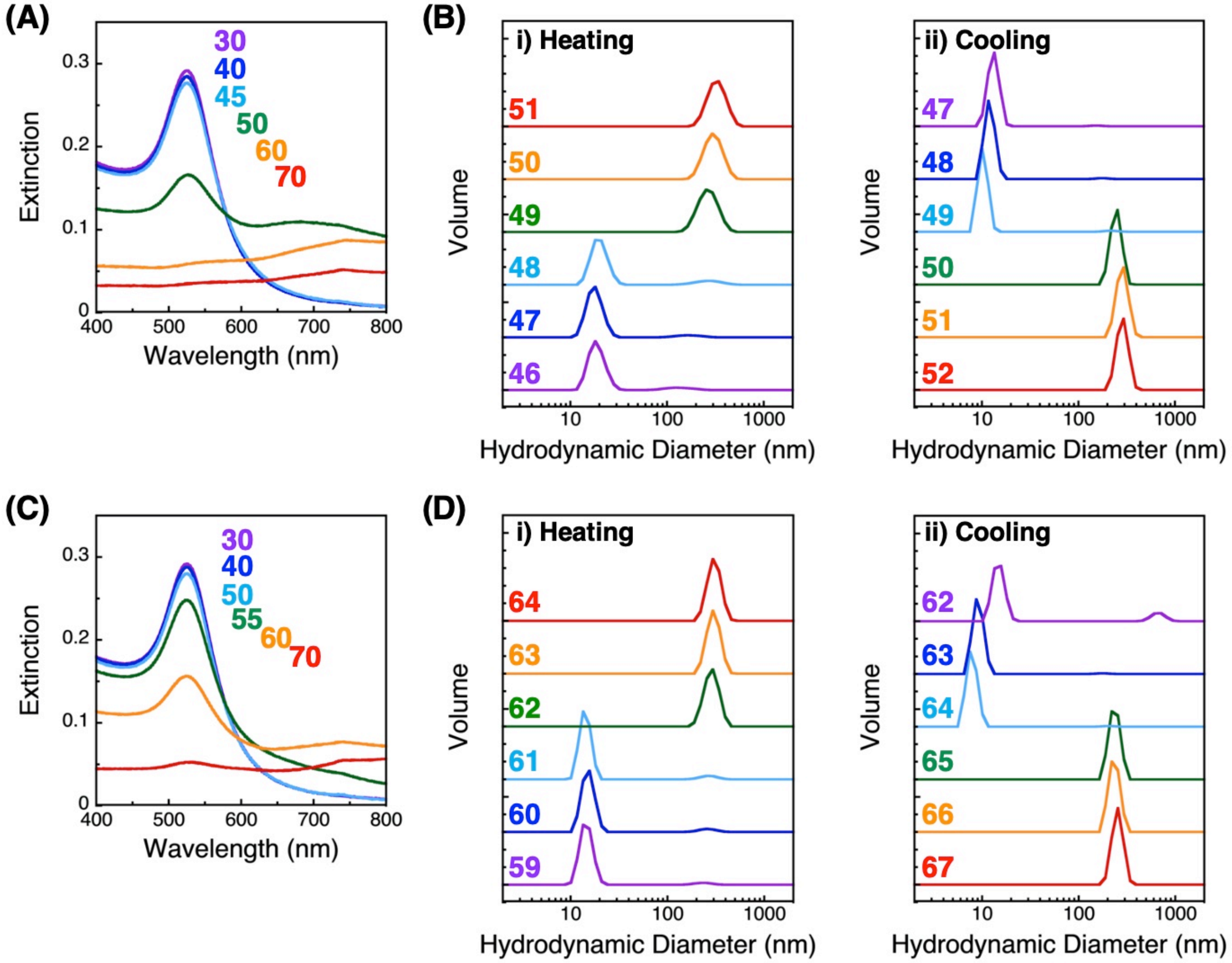

Figure S7. Extinction spectra (A)(C) and size distribution (B)(D) of 15-nm AuNPs coated with a mixture of **C2-EG6** and **HO-EG6** ligands. (A)(B) AuNPs modified with **C2-EG6** (95%) + **HO-EG6** (5%). (C)(D) AuNPs modified with **C2-EG6** (90%) + **HO-EG6** (10%).

Table S4. Hydrodynamic diameter and zeta-potential of AuNPs before and after surface modification with a mixture of **C2-EG6** and **HO-EG2** ligands.

**C2-EG6 : HO-EG2 Ligand Ratio**	Zeta-Potential (mV)	S.D.	Size*[1] (nm)	S.D.
80 : 20	-5.2	2.1	18.4	0.8
60 : 40	-5.4	1.3	20.9	5.8
40 : 60	-5.0	1.5	17.6	0.3
20 : 80	-7.3	0.4	17.7	0.5

*[1] Size was determined as the main peak size in the DLS size distribution by volume.

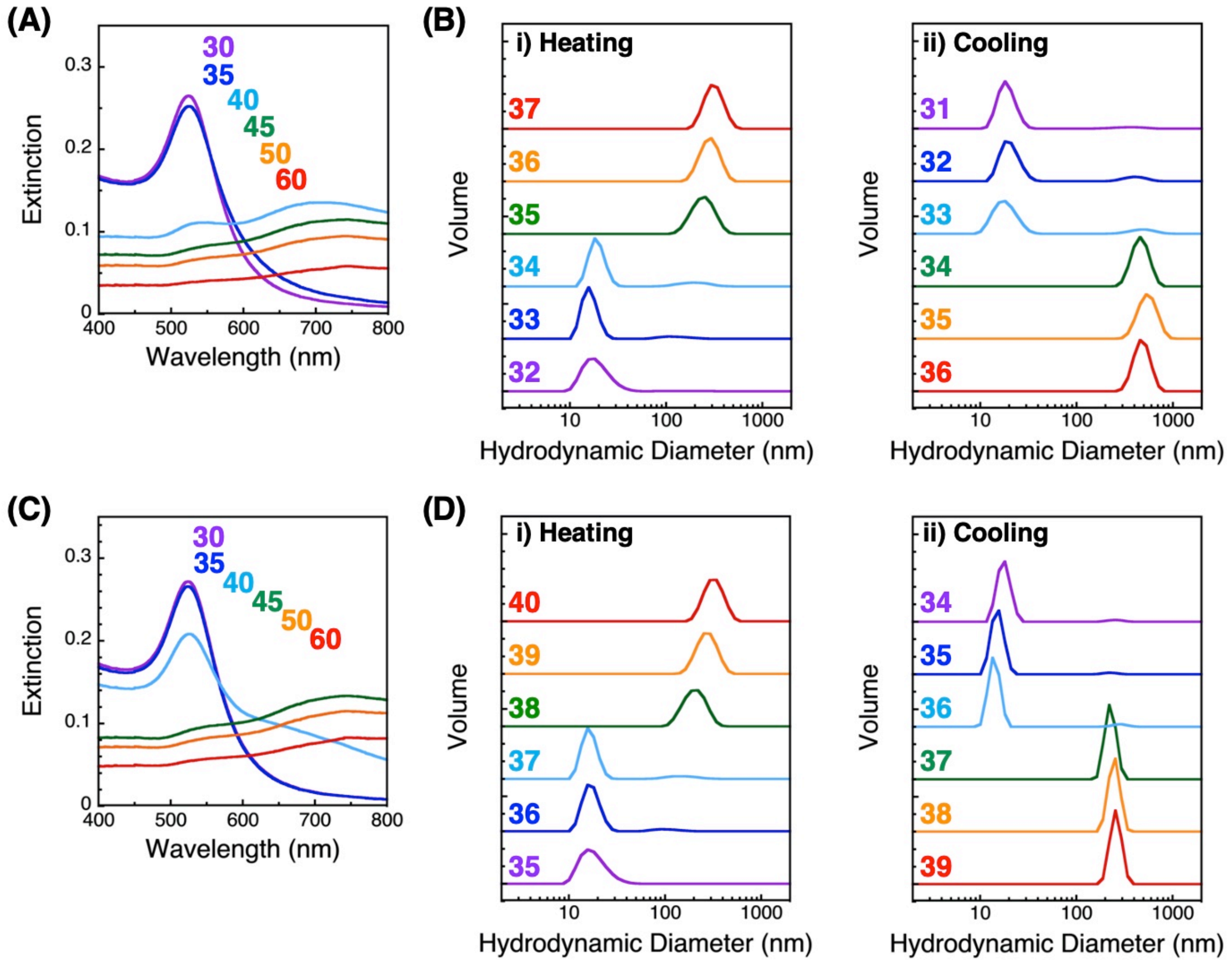

Figure S8. Extinction spectra (A)(C) and size distribution (B)(D) of 15-nm AuNPs coated with a mixture of **C2-EG6** and **HO-EG2** ligands. (A)(B) AuNPs modified with **C2-EG6** (80%) + **HO-EG2** (20%). (C)(D) AuNPs modified with **C2-EG6** (60%) + **HO-EG2** (40%).

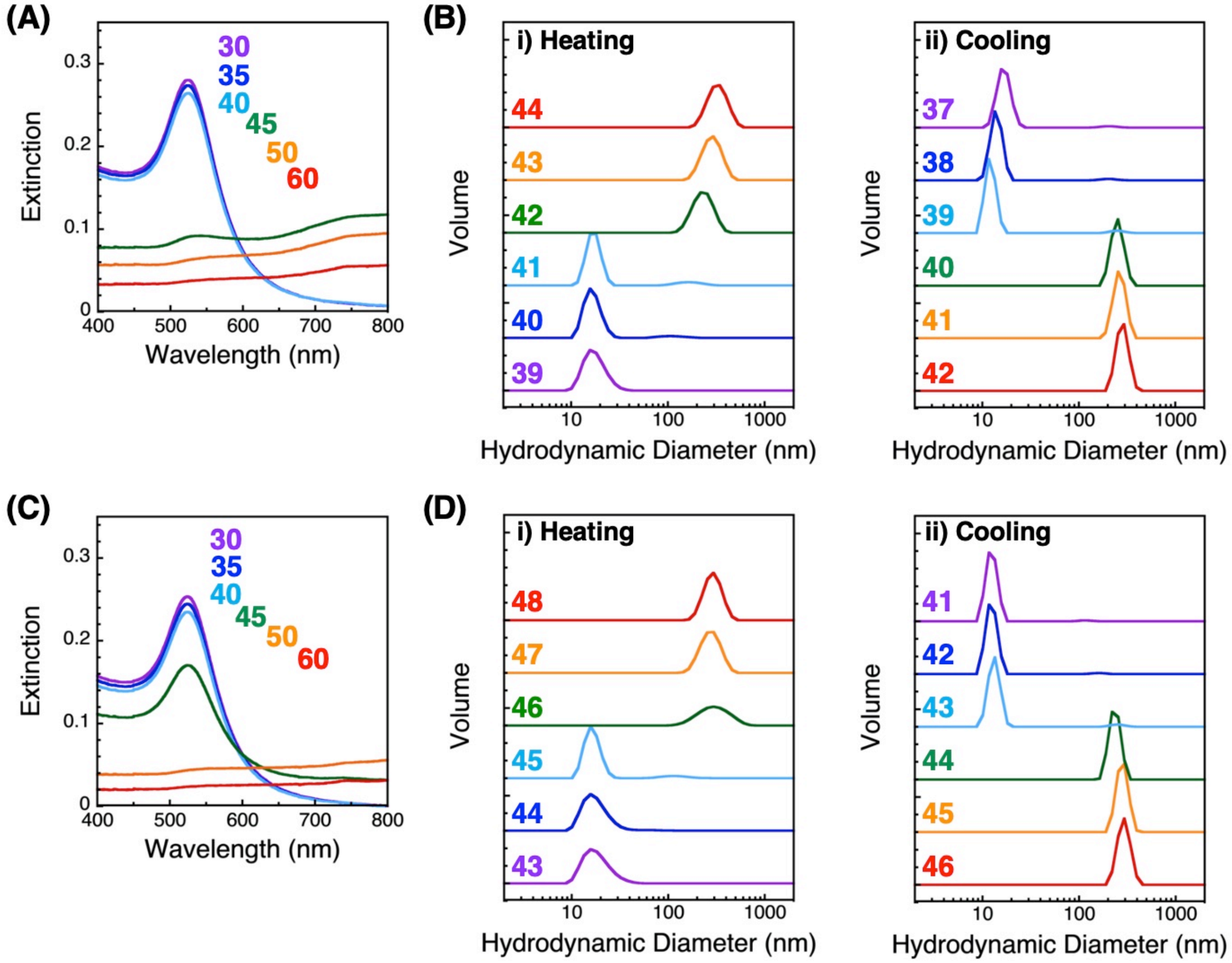

Figure S9. Extinction spectra (A)(C) and size distribution (B)(D) of 15-nm AuNPs coated with a mixture of **C2-EG6** and **HO-EG2** ligands. (A)(B) AuNPs modified with **C2-EG6** (40%) + **HO-EG2** (60%). (C)(D) AuNPs modified with **C2-EG6** (20%) + **HO-EG2** (80%).

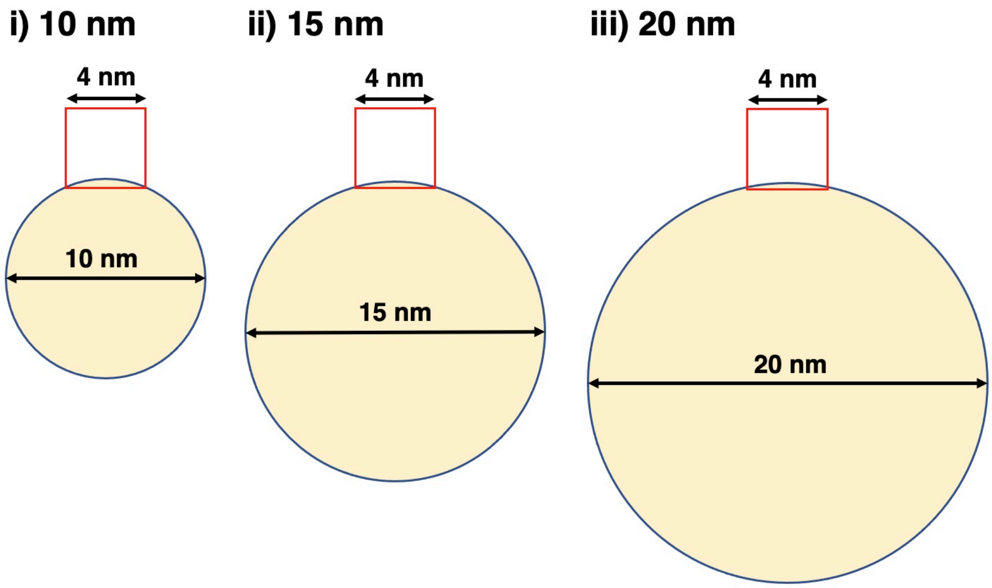

Figure S10. Schematics of the MD simulation box for curved surfaces on (i)10-, (ii) 15-, and (iii) 20-nm AuNP.

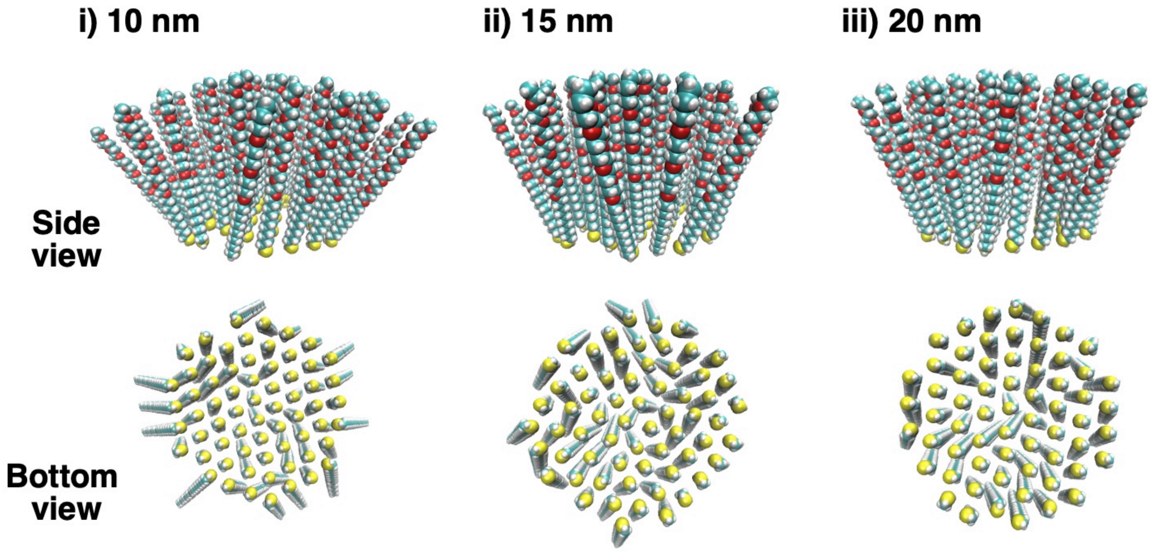

Figure S11. Initial configuration of **C2-EG6** ligands planted on the sphere cap with $2R$ = 10 (i), 15 (ii), and 20 nm (iii).

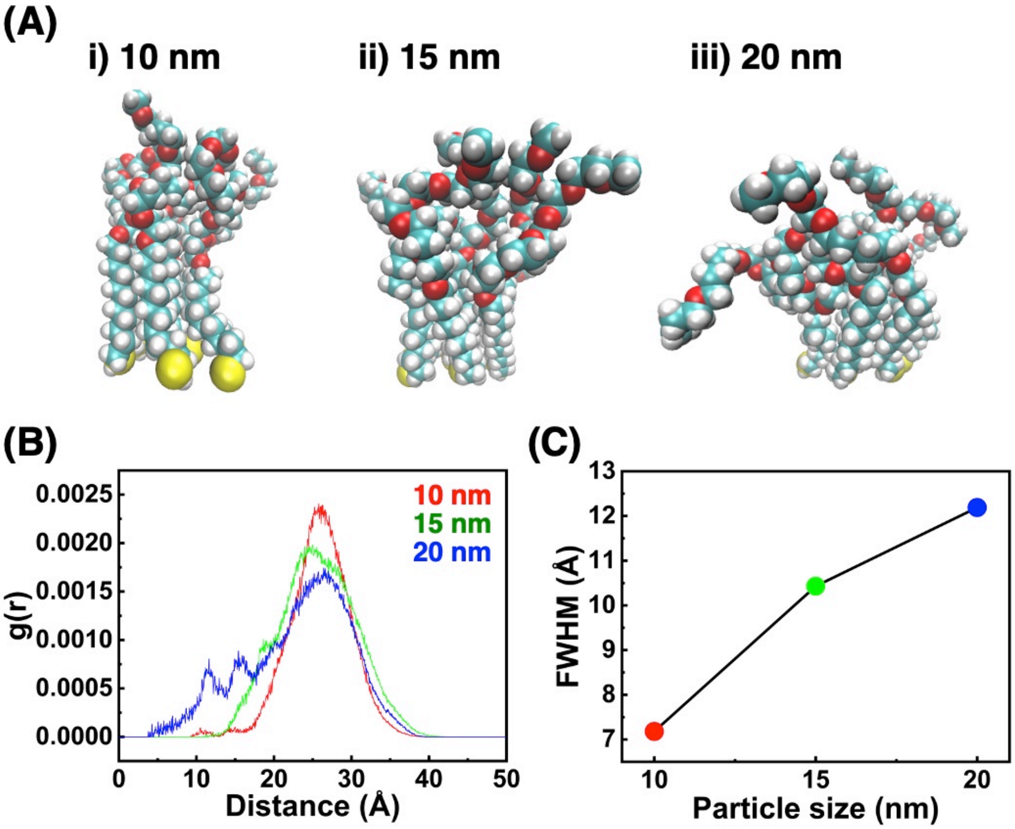

Figure S12. (A) Snapshots of seven **C1-EG6** ligands in the vicinity of the surface center of 10- (i), 15- (ii), and 20-nm AuNPs (iii), (B) the averaged RDF of the seven terminal carbon atoms against the corresponding sulfur atoms around the center of the gold surface, and (C) their FWHM for each AuNP diameter.

Ligand mixing ratios C2-EG6 : HO-EG2	Initial state	Simulated state
100 : 0	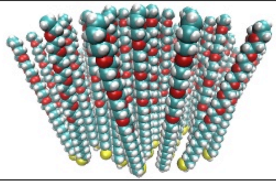	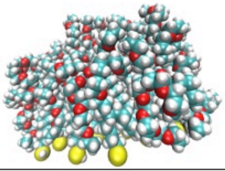
80 : 20	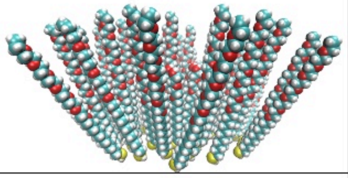	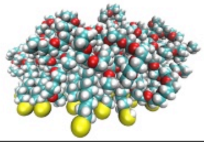
60 : 40	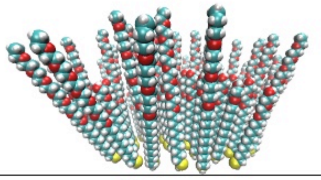	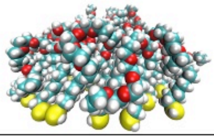
40 : 60	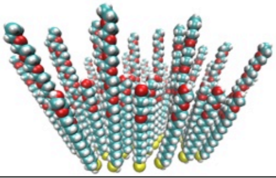	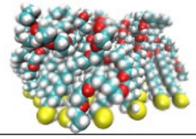
20 : 80	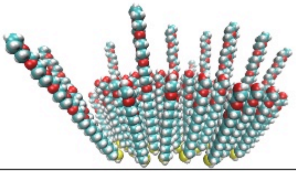	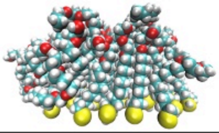

Figure S13. Side views of **C2-EG6** and **HO-EG2** ligand mixtures planted on the sphere cap with $2R$ = 15 nm as an initial configurations and snapshots of simulated configurations.

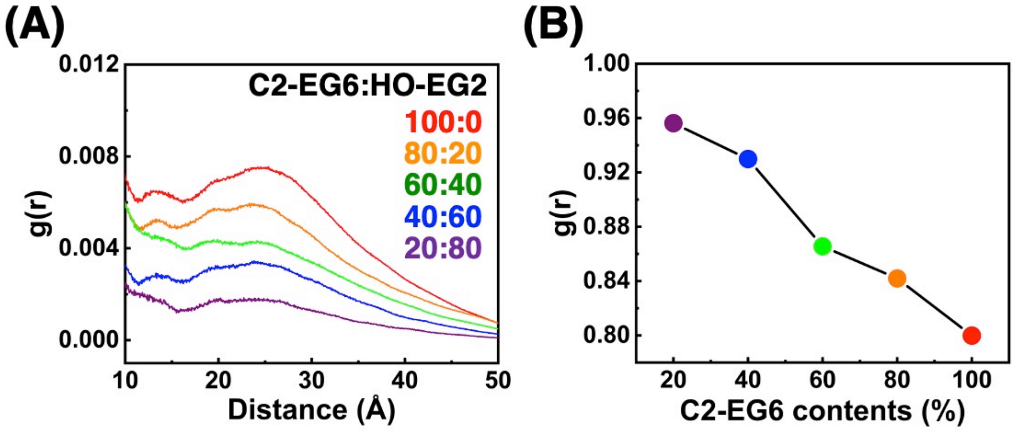

Figure S14. (A) The RDF of all the terminal carbon atoms against the corresponding sulfur atoms attached on gold surfaces and (B) the intensity plots of the first water-solvation shell of the terminal on a 15-nm AuNP against varying mixing ratios of **C2-EG6** and **HO-EG2**.

References

(1) Camillone, N.; Leung, T. Y. B.; Schwartz, P.; Eisenberger, P.; Scoles, G. Chain Length Dependence of the Striped Phases of Alkanethiol Monolayers Self-Assembled on Au(111): An Atomic Beam Diffraction Study. *Langmuir* **1996**, *12* (11), 2737–2746.

(2) Iida, R.; Mitomo, H.; Matsuo, Y.; Niikura, K.; Ijiro, K. Thermoresponsive Assembly of Gold Nanoparticles Coated with Oligo(Ethylene Glycol) Ligands with an Alkyl Head. *J. Phys. Chem. C* **2016**, *120* (29).
